# Metabolic Profiles of *Brassica juncea* Roots in Response to Cadmium Stress

**DOI:** 10.3390/metabo11060383

**Published:** 2021-06-13

**Authors:** Piaopiao Tan, Chaozhen Zeng, Chang Wan, Zhe Liu, Xujie Dong, Jiqing Peng, Haiyan Lin, Mei Li, Zhixiang Liu, Mingli Yan

**Affiliations:** 1 Hunan Provincial Key Laboratory of Forestry Biotechnology, College of Life Science and Technology, Central South University of Forestry and Technology, Changsha 410004, China; piaopiaotanznl@163.com (P.T.); chaozhenzeng@163.com (C.Z.); changwan0129@163.com (C.W.); lstlz1121@163.com (Z.L.); dxj801230@163.com (X.D.); pengjiqing17@cusft.edu.cn (J.P.); 2 Hunan Provincial Base for Scientific and Technological Innovation Cooperation on Forest Resource Biotechnology, Changsha 410004, China; 3 Hunan Provincial Key Laboratory of Crop Germplasm Innovation and Utilization, Hunan Agricultural University, Changsha 410128, China; cuiyarong1015@126.com; 4 Crop Research Institute, Hunan Academy of Agricultural Sciences, Changsha 410125, China; limei1230@126.com; 5 Hunan Key Laboratory of Economic Crops Genetic Improvement and Integrated Utilization, Hunan University of Science and Technology, Xiangtan 411201, China

**Keywords:** *Brassica juncea*, cadmium stress, pathway analysis, metabolite profiling

## Abstract

*Brassica juncea* has great application potential in phytoremediation of cadmium (Cd)-contaminated soil because of its excellent Cd accumulating and high biomass. In this study, we compared the effects of Cd under 48 h and 7 d stress in roots of *Brassica juncea* using metabolite profiling. The results showed that many metabolic pathways and metabolites in *Brassica juncea* roots were altered significantly in response to Cd stress. We found that significant differences in levels of amino acids, organic acids, carbohydrates, lipids, flavonoids, alkaloids, and indoles were induced by Cd stress at different times, which played a pivotal role in the adaptation of *Brassica juncea* roots to Cd stress. Meanwhile, *Brassica juncea* roots could resist 48 h Cd stress by regulating the biosynthesis of amino acids, linoleic acid metabolism, aminoacyl-tRNA biosynthesis, glycerophospholipid metabolism, ABC transporters, arginine biosynthesis, valine, leucine and isoleucine biosynthesis, and alpha-linolenic acid metabolism; however, they regulated alpha-linolenic acid metabolism, glycerophospholipid metabolism, ABC transporters, and linoleic acid metabolism to resist 7 d Cd stress. A metabolomic expedition to the response of *Brassica juncea* to Cd stress will help to comprehend its tolerance and accumulation mechanisms of Cd.

## 1. Introduction

Heavy metals contamination in cultivated soil caused by anthropogenic activities such as mining, smelting, manufacturing, pesticides, sewage sludge, and sewage irrigation has become an increasingly serious environmental problem both for human health and agriculture industry in recent decades [1]. The major toxic heavy metals that have been found around the world include cadmium (Cd), arsenic (As), and lead (Pb) [2,3,4]. Among these, Cd is the most dangerous environmental pollutant for its toxic and mutagenic effects on both plants and animals [5]. In addition, Cd is easily absorbed by plants and then transferred into the human bodies through the food chain, which increases the risk to public health [6]. The toxic effects of Cd on plants have been widely studied. Cd accumulation not only inhibits plant growth [7] but also causes a variety of physiological and biochemical changes [8]. After entering the plant body, Cd can disturb photosynthetic activity, chlorophyll synthesis, water relations, mineral uptake, inhibit a variety of metabolic processes, and induce oxidative stress [9,10].

*Brassica juncea* belonging to the family *Brassicaceae* is a high-yielding, fast-growing double diploid plant, and is one of the major crops in China [11]. *B. juncea* can accumulate Cd, Cu, Cr, Zn, Ni, Au, Se, and other heavy metals from contaminated soil due to its high capacity of metal enrichment, thereby it is regarded as a plant that can “extract” heavy metal [12]. Compared with other heavy metal-accumulating plants, *B. juncea* has unique characteristics of heavy metal enrichment [13]. Accumulated evidence shows that *B. juncea* has specific physiological mechanisms and structural characteristics, which can contribute to the adaptation of high-concentration heavy metal environment and accumulate harmful heavy metal ions in vivo [14,15]. Therefore, it is of great significance to understand the toxicity or detoxification mechanism of Cd from the perspective of optimizing Cd phytoremediation by using *B. juncea*.

Plant metabolomics aims to help understand the basic metabolic mechanism of plants when responding to environmental changes or gene mutations [16,17]. *B. juncea* would be a good candidate to study the metabolic changes of secondary metabolites, understand metabolic networks, and explore the possible mechanism of tolerance or hyperaccumulation of Cd in Cd-rich plants [18]. Luo et al. [19] studied the metabolic spectrum changes of exudates in *Sedum alfredii* root under Cd stress by using the metabolomic method based on gas chromatography-mass spectrometry (GC-MS) and found 12 root exudates with significant differences under different Cd treatment. Navarro-Reig et al. [20] analyzed the metabolite changes of *Oryza sativa* L. under Cd and Cu stress by using LC-MS, combined with multivariate statistical methods, and identified 97 metabolites with significant differences. Compared with GC-MS, LC-MS has higher peak capacity, resolution, and sensitivity, and is suitable for the analysis of metabolites with high boiling point, high molecular weight, or limited thermal stability; therefore, LC-MS is more suitable for the detection of various metabolites [21]. Metabolites associated with aspects of the detoxification of heavy metal have been reported including organic acids, amino acids, peptides, glutathione, and phytochelatins [14]. However, the underlying metabolic mechanisms are unclear.

In this study, a non-targeted metabolomics approach based on LC-MS was applied to investigate the characteristics of *B. juncea* under Cd stress. Multivariate statistical analysis, including principal component analysis (PCA) and orthogonal partial least-squares-discriminant analysis (OPLS-DA), were exploited to evaluate the metabolomic profiling, analyze metabolic pathways of different metabolites, and explore the possible mechanism of Cd toxicity of *B. juncea*.

## 2. Results and Discussion

### 2.1. Changes of Cd Content under Cd Stress at Different Times

**Figure 1:**
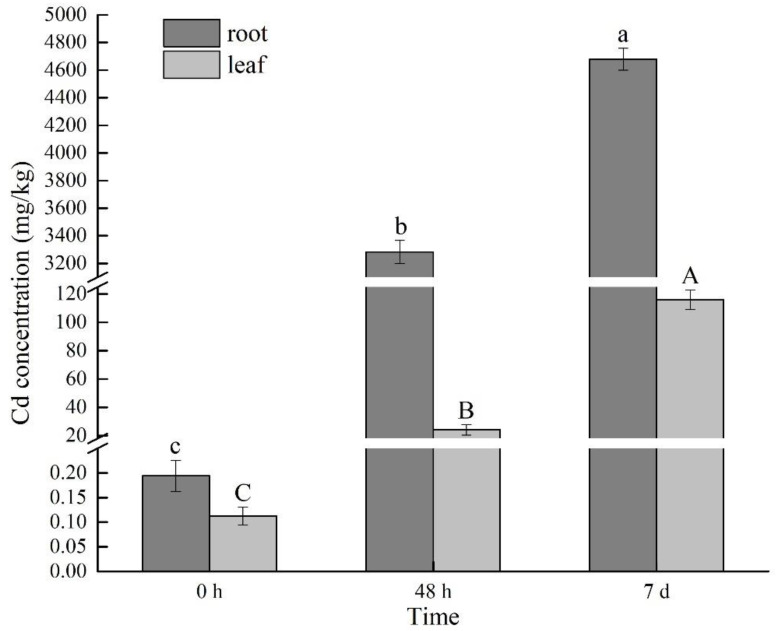
Changes of Cd content in *Brassica juncea* at different Cd stress times. The analysis was performed with three biological replicates. Error bars indicate the standard error of the mean (SEM). 0 h (T1), 48 h (T2), and 7 d (T3). Means with different letters for each treatment are significantly different at *p* < 0.05 by the LSD test. Capital letters (**A**–**C**) and lower-case letters (**a**–**c**) show significantly different of Cd content in leaf and root, respectively.

As shown in Figure 1, the Cd content in *B. juncea* roots showed an increasing trend with the increase of Cd stress time, and the longer the stress time was, the higher the Cd content was. After seven days of Cd treatment, the Cd content in the leaves of *B. juncea* exceeded 100 mg/kg DW (Figure 1), and this value was considered to be the threshold for Cd hyperaccumulators [22]. Therefore, this inbred line of *B. juncea* has a strong ability to accumulate Cd. The Cd content in roots was significantly higher than that in leaves, and the translocation index (TI) of Cd at 48 h and 7 d was 0.007 and 0.025, respectively. This indicated that most of the heavy metals absorbed by *B. juncea* were accumulated in the roots, and a small part was transported to the leaves, which was similar to the results of Ahmad et al. [23].

### 2.2. Changes of Biomass under Cd Stress at Different Times

**Figure 2:**
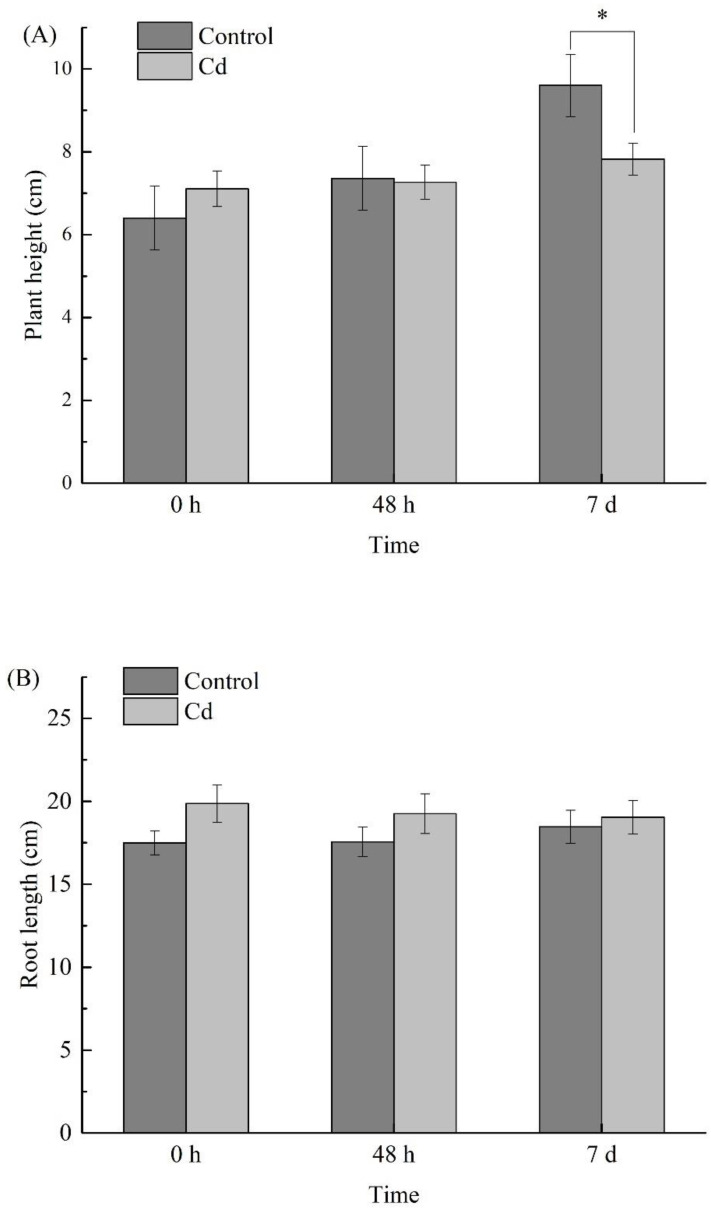
Changes of plant height (**A**) and root length (**B**) in *Brassica juncea* at different Cd stress times. The analysis was performed with 30 biological replicates. Error bars indicate the standard error of the mean (SEM). The asterisks show statistically significant difference between the means at *p* < 0.05 (*), 0 h (T1), 48 h (T2), and 7 d (T3). The differences at *p* < 0.05 (*) are shown to be significant by the LSD test.

**Figure 3:**
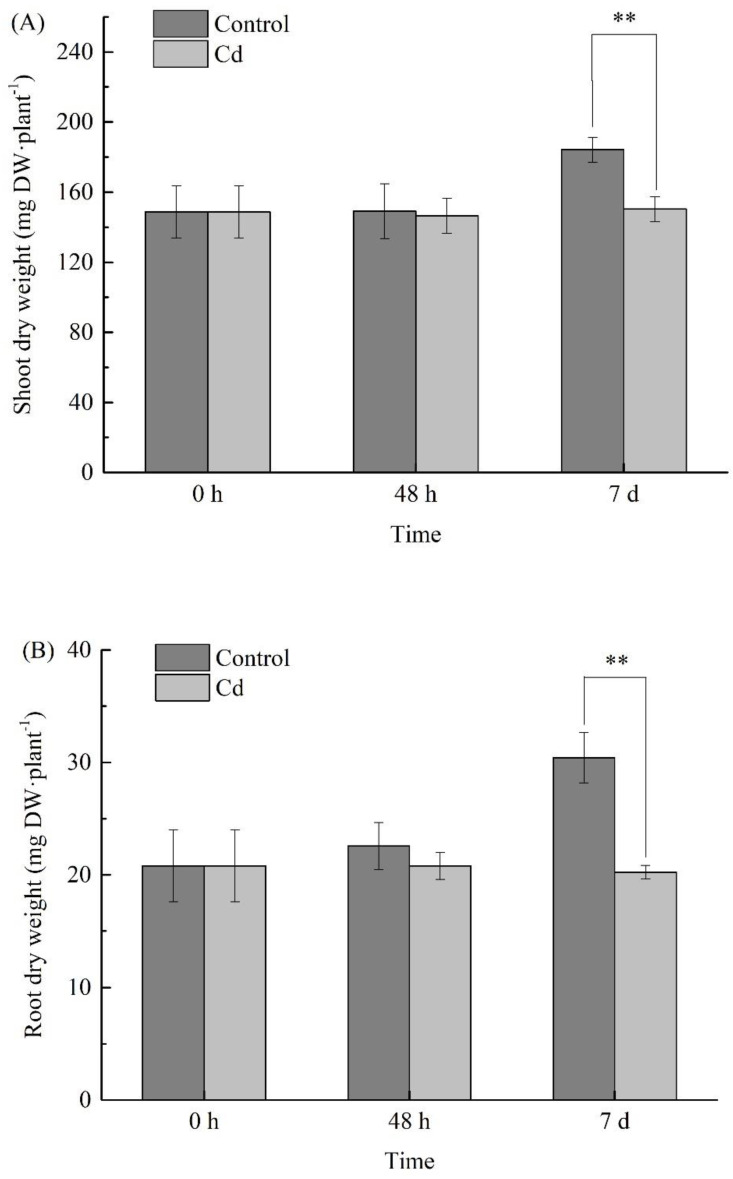
Changes of biomass in *Brassica juncea*. Dry biomass of shoot (**A**) and root (**B**) were determined with 30 biological replicates. Error bars indicate the standard error of the mean (SEM). The asterisks show statistically significant difference between the means at *p* < 0.01 (**), 0 h (T1), 48 h (T2), and 7 d (T3).

There was no significant difference in the plant height and root length of *B. juncea* between the treated group and the control group after 48 h and 7 d of Cd stress (Figure 2). But in terms of plant dry weight (Figure 3), compared with the control, the growth of *B. juncea* was inhibited significantly (*p* < 0.01) after 7 days of Cd stress.

**Figure 4:**
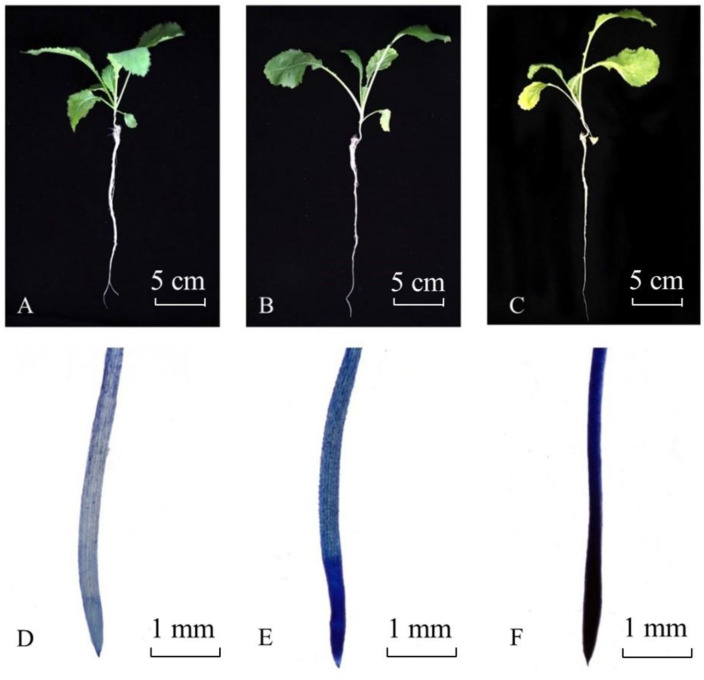
Plants and Evans blue stained root tips of *Brassica juncea* at different Cd stress times. (**A**,**D**) (T1): 0 h; (**B**,**E**) (T2): 48 h; (**C**,**F**) (T3): 7 d.

No significant symptoms were observed in *B. juncea* plants under 48 h Cd stress, whereas 7d Cd stress caused chlorosis of leaves (Figure 4A–C). Stress induced cell death or injury in root tip can be detected by Evans blue staining [24]. As shown by Evans blue staining, a large number of cells were damaged (blue) at the root tip of Cd treated plant, and cell death was more serious at the meristematic zone under Cd stress (Figure 4D–F). Moreover, with longer Cd-stressed time, the damage of cells became more serious.

### 2.3. Nontargeted Analysis of B. juncea by LC-MS

The base peak chromatogram (BPC) was determined for the *B. juncea* samples (T1, T2, and T3) in the positive and negative ionization modes. Metabolites demonstrated significant differences between the T1, T2, and T3 samples (Supplemental Figures S1 and S2). The *B. juncea* metabolome was separated on an ACQUITY UPLC BEH C18 column within 16 min using LC-MS. In total, 6789 and 1174 metabolites were detected from data sets of LC-MS in the positive and negative ionization modes, respectively.

### 2.4. Multivariate Analysis in Different B. juncea Samples

Unsupervised principal component analysis (PCA) was used to observe the stability of the experiment analysis process. As depicted in Figure S3, the QC samples cluster closely compared to the total variance in the experiment, which revealed that the dataset had high stability and repeatability. Figures S4A and S5A showed that PC1 and PC2 were successfully separated in *B. juncea* samples, revealing that the metabolic profiles of the *B. juncea* roots under Cd stress at different times were significantly different. The results showed that the two principal components (PCs) explained 62% (T2/T1 comparative group) (Figure S4A) and 52.2% (T3/T1 comparative group) of variance (Figure S5A). Supervised PLS-DA and OPLS-DA were used to distinguish differences in metabolic profiles and find differential metabolites between groups. As shown in Figures S4B and S5B, the R^2^Y, and Q^2^ values of the PLS-DA were 0.989 and 0.905 in T2/T1 comparative group, and 0.996, 0.944 in T3/T1 comparative group, respectively. The R^2^Y and Q^2^ values of the OPLS-DA were 0.97 and 0.876 in T2/T1 comparative group, and 0.996 and 0.944 in T3/T1 comparative group, respectively (Figures S4C and S5C). To prevent the OPLS-DA model from overfitting, the methods of 7-fold cross validation and 200 response permutation testing (RPT) were applied to examine the quality of the model. The R^2^ and Q^2^ intercepts of cross validation were 0.79 and −0.482 in T2/T1 comparative group, and 0.922 and −0.373 in T3/T1 comparative group, respectively (Figures S4D and S5D). These results showed the models were reliable and stable.

### 2.5. Differential Metabolites Analysis

The multivariate and univariate results were integrated to acquire the criteria for screening the differential metabolites, selecting the differential metabolites between T2 and T1, T3, and T1 [25]. Differential metabolites of *B. juncea* roots were selected when VIP > 1 and *p*-value < 0.05. Fold change (FC) represents the ratio of the average contents of metabolites in two groups of samples. Differential metabolites with Log_2_ (FC) > 0 indicate upregulation, and Log_2_ (FC) < 0 indicate downregulation. The differential metabolites included amino acids, organic acids, carbohydrates, lipids, flavonoids, etc. (Supplemental Tables S1 and S2).

#### 2.5.1. Amino Acids

As illustrated in Tables S1 and S2, 54 (33 upregulated; 21 downregulated), 66 (31 upregulated; 35 downregulated) different amino acids and their derivatives were identified in the T2/T1 and T3/T1 groups, respectively. We found that 48 h and 7 d Cd treatment could stimulate up or downregulation of some amino acids and their derivatives, and there were great differences in varieties and quantities in the roots of *B. juncea* (Tables S1 and S2). After 48 h Cd stress, L-aspartic acid, L-lysine, L-arginine, L-glutamate, beta-tyrosine, and L-isoleucine increased significantly in the roots of *B. juncea*. Under 7 d Cd stress, the content of L-lysine, L-histidine, and glutathionate increased significantly, while the contents of beta-tyrosine and glutamine (D, L) decreased significantly.

Amino acids and their derivatives have been reported to form complexes with heavy metal ions primarily via carboxylate (–COO) and amine (–NH_2_) groups, thus conferring plants with heavy metal tolerance [26,27]. It has been reported that Cd resulted in the increases of asparagine, methionine, and lysine in lettuce [28]. Under drought stress, the accumulation of asparagine and glutamine can reabsorb free amino acids released and contribute to the reduction in the toxic effect of ammonia salts on plants [29]. In this study, N-oleoyl asparagine was significantly increased after Cd treatment (7 d), but did not change significantly under 48 h Cd stress. The content of L-lysine was significantly increased in both T2/T1 and T3/T1. Similar results were reported in *Sargassum fusiforme* leaf under heat stress [30]. Amino acid, particularly proline was a widely studied molecule in the context of plant responses to abiotic stresses. Many plants accumulated proline under water deficit [31], salt stress [32], low temperature stress [33], and some other abiotic stresses. As depicted in Tables S1 and S2, upregulation of most proline derivatives was encountered under Cd stress, too. The accumulation of some proline derivatives could improve the abiotic stress resistance in plants [34].

Tyrosine, an aromatic amino acid, plays a pivotal regulatory function during development and defense responses in plants. It was accumulated in Cd-stressed shoots of Compositae plants and reduced its antioxidant damage [35]. In the present experiment, beta-tyrosine content of *B. juncea* roots was increased under 48 h Cd stress, in contrast, decreased under 7 d stress. Similar results were reported by Cosio and Renault [36] that tyrosine content of *Elodea nuttallii* was significantly increased after 24 h of Cd treatment. This suggests that tyrosine may be involved in response to 48 h Cd stress and reduce antioxidant damage in *B. juncea* through its accumulation.

Branched amino acids (i.e., leucine, isoleucine, and valine) are considered as precursors for some secondary metabolites, such as alkaloids and glycosides, and participate in both biotic and abiotic stress responses [36]. Previous studies reported that levels of isoleucine and valine were accumulated in *Solanum nigrum* roots [37] and *E. nutalli* shoots [36] under Cd stress. In this paper, L-isoleucine was significantly upregulated at T2/T1, but not significantly changed at T3/T1. In comparison with 7 d Cd stress, the concentration of L-isoleucine was higher under 48 h Cd stress. Valine, leucine, and isoleucine biosynthesis can withstand osmotic imbalance in response to heavy metals stress [38], and was enriched under short-term Cd stress in *B. juncea* roots, which interpreted the variation of L-isoleucine content in our present study.

Histidine can regulate the synthesis of some amino acids and is involved in chelating and transporting metal ions, thereby playing a vital role in heavy metal stress [39]. Our study found that L-histidine content was significantly improved together with increasing Cd concentrations. Similarly, the results were reported in the leaves of *Compositae* plants exposed to Cd [35].

Glutamate is a precursor of several amino acids closely related to stress, including proline, arginine, and γ-aminobutyric acid. It had been shown that glutamate was involved in response to heavy metal stresses; in addition, exogenous glutamate, but not glutamine, significantly mitigated Cd toxicity in rice [40]. As shown in Tables S1 and S2, L-glutamate was increased significantly in T2/T1, glutamine (D, L) was decreased significantly in T3/T1. Thereby, compared with glutamine, L-glutamate plays a more important role in Cd tolerance of *B. juncea*. Arginine is a precursor of polyamines, which plays a pivotal function in plant adversity-resistance [41]. Arginine biosynthesis was enriched under 48 h Cd stress, which interpreted the variation of L-arginine content in our present study.

#### 2.5.2. Organic Acids

Organic acids secreted by plant roots can reduce the toxicity of heavy metals by changing their forms, thereby promoting the uptake of heavy metals by plants [42]. Furthermore, heavy metals can be chelated with low molecular weight organic acids stored in vacuoles and transformed into low or non-toxic chelating states to help plants resist the stress, which is a common mechanism of heavy metal defense system [43]. In the present study, we detected 16 and 14 organic acids in T3/T1 and T2/T1 comparative groups, respectively, most of which were upregulated. It has also been reported that the formation of Cd-organic acid complex stimulated to absorb more Cd in maize roots [44]. An increase of oxalic acid dibutyl ester content was found in both T2/T1 and T3/T1, and the increase was more in T3/T1. This indicated that its content increased together with the increase of Cd content in *B. juncea* roots. revealed that oxalic acid content was increased with increasing Cd concentration in the rhizosphere of maize cultivars (cv. 3062). Previous research has demonstrated that organic acids such as citric acid and malic acid form stable metal-ligand complexes with heavy metal ions in plants and participate in the separation of ions in vacuoles [45]. In this study, compared with the control, triethyl citrate was decreased significantly 48 h after Cd treatment, but there was no significant difference under 7 d Cd stress.

#### 2.5.3. Carbohydrates

Carbohydrates such as hexoses (glucose), disaccharides (trehalose), and oligosaccharides (maltose) are important compatible osmotic substances that assisted plants in resisting stress [46,47]. There have been numerous reports of carbohydrate accumulation under water deficit [48], salinity [49], low temperature [47], or osmotic stress [50]. In this study, 23 and 45 carbohydrates were observed in T3/T1 and T2/T1, respectively, such as trehalose, lactose, maltose, sucrose, maltotriose, rhamnose, and so on. These carbohydrates were upregulated more at T3/T1 than at T2/T1 groups (Tables S1 and S2). It was also indicated that in comparison with 48 h stress, more carbohydrates were accumulated to resist 7 d Cd stress of *B. juncea*. It was shown that *Bermudagrass* accumulated a variety of sugars under Cd stress, especially fructose, galactose, glucose, trehalose, and heptanose [51]. Krasavina et al. [52] reported that the accumulation of trehalose under abiotic stress helped to improve plant resistance. Guy et al. [47] concluded that, under drought and high temperature stress, plants accumulated several soluble sugars, including maltose, trehalose, and glucose. Consistent with previous studies, our results suggest that carbohydrates play important roles in the response of *B. juncea* roots to Cd stress, especially to 7 d stress.

#### 2.5.4. Lipids

The first functional structure in plant cells to be exposed to toxic metals is the plasma membrane, which played a crucial role in plant metal tolerance. Lipids are a group of fats and fat-like substances that are necessary to maintain the structural integrity of membranes. Lipids have a significant effect in adjusting adversity-resistant of plant cells. The role of lipids in metal stress has been demonstrated [53]. In the present study, we detected 71 (44 upregulated, 27 downregulated; T2/T1 samples) and 72 (18 upregulated, 54 downregulated; T3/T1 samples) lipids that exhibited statistically significant differences in concentrations. Compared with T2/T1, there were more lipids significantly downregulated in the T3/T1. Therefore, it was inferred that most of lipids were significantly decreased under 7 d Cd exposure. In a previous study, it was found that the content of sitosterol decreased under Cd, mercury (Hg), and aluminum (Al) stress [54,55]. Huynh et al. [56] proved that the longer the rice was under Al stress, the lower the total lipid content in its roots and shoots. In root exudates of *Sedum plumbizincicola* exposed to Cd, the lipids concentration was significantly decreased [57].

#### 2.5.5. Flavonoid Glycosides, Alkaloids, and Indoles

It is known that flavonoids are secondary metabolites with strong oxidation resistance, thereby mainly removing reactive oxygen species (ROS) under adversity stress in plants [58]. Meanwhile, flavonoids and metal ions can form metal-ligand complexes, which reduce metal phytotoxicity [58]. As shown in Tables S1 and S2, 9 and 10 flavonoid glycosides were identified in T2/T1 and T3/T1 comparative groups, respectively, and most of them were upregulated. This suggests that flavonoids play a cardinal role in the resistance of *B. juncea* subjected to Cd stress. In the Cd-stressed *Amaranthus Hypochondriacus,* seven flavonoids—including kaempferol, rutin, and quercetin—were found to change significantly [38]. It was revealed that flavonoids might be resisted Cd-induced damage in *naphthalene hydroxylase G*-transformed line (*nahG*) roots [59] and Brazilian elodea shoots [58].

Alkaloids are secondary metabolites in response to biotic and abiotic stresses in plants [60]. The data showed that most of alkaloids showed upregulation in T2/T1 and T3/T1 samples. Therefore, we believed that significantly high alkaloids were accumulated in Cd-stressed *B. juncea* roots. Similar results were also reported by Srivastava and Srivastava [61] in *Catharanthus roseus* L.

It was witnessed that indoles growth regulators, such as indole-3-acetic acid (IAA) and melatonin, could mitigate heavy metal toxicity in plants [62,63]. Indoles were all downregulated in T3/T1, but some upregulated and others downregulated in T2/T1. Camalexin is prepared by indole-3-acetaldoxime to resist pathogen attack or heavy metal toxicity [64]. Pedras et al. [65] reported that camalexin could enhance the detoxification capacity of the phytoalexin brassinin to resist *Leptosphaeria maculans.* Interestingly, we observed that the concentration of camalexin significantly decreased with the extension of Cd stress time compared with the control (Tables S1 and S2). It was inferred that camalexin content might be affected by cadmium toxicity, and a negative correlation between Cd and camalexin content has been found in *B. juncea* roots.

### 2.6. Differential Metabolic Pathways and Metabolic Network Analyses

**Figure 5:**
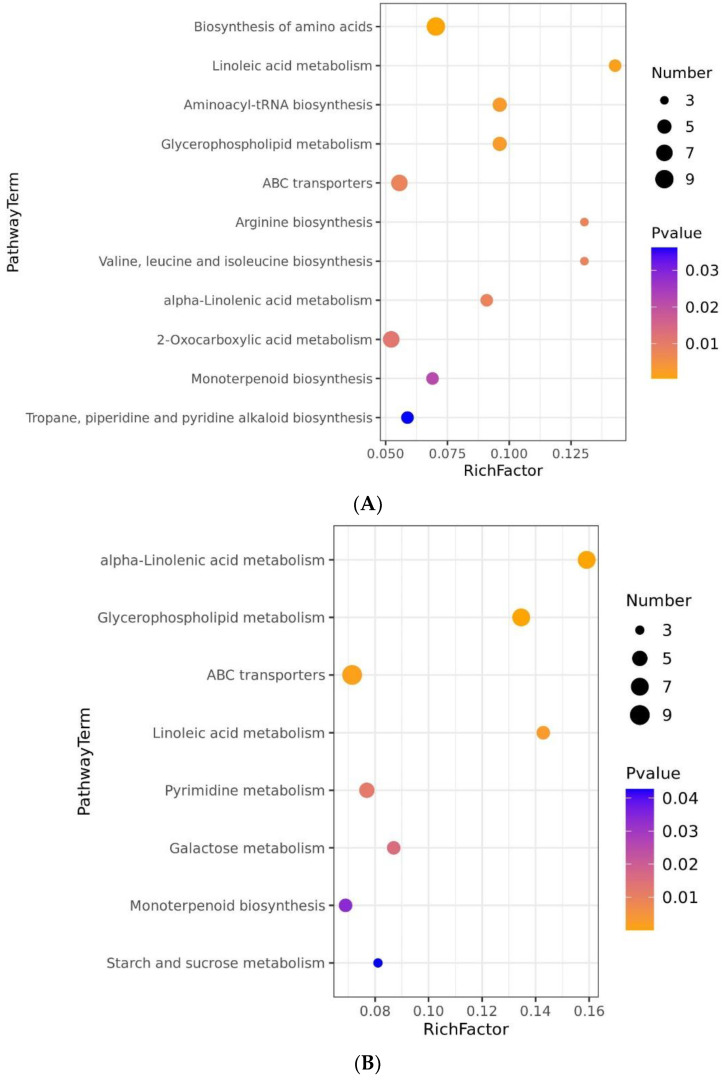
KEGG enrichment analysis of differential metabolic pathways in *Brassica juncea* samples. (**A**) Differential metabolic pathways enrichment in T2/T1 samples; (**B**) T3/T1 samples. The vertical coordinate represents the name of the metabolic pathway, and the horizontal coordinate represents the enrichment factor (Rich factor, number of significantly different metabolites/total number of metabolites in the pathway). The greater the Rich factor, the greater the degree of enrichment. A color-coded bar on the right indicates the size of the *p*-value. The color changes from orange to blue, indicating the *p*-value decreases in order. The points represent the number of metabolites. The larger the point, the more metabolites were enriched into the pathway.

The KEGG pathways, mainly including biosynthesis of amino acids, linoleic acid metabolism, aminoacyl-tRNA biosynthesis, glycerophospholipid metabolism, ABC transporters, arginine biosynthesis, valine, leucine, and isoleucine biosynthesis, and alpha-linolenic acid metabolism, were disturbed under 48 h Cd stress (Figure 5A). However, after 7 d Cd stress, alpha-linolenic acid metabolism, glycerophospholipid metabolism, ABC transporters, and linoleic acid metabolism were disturbed (Figure 5B).

**Figure 6:**
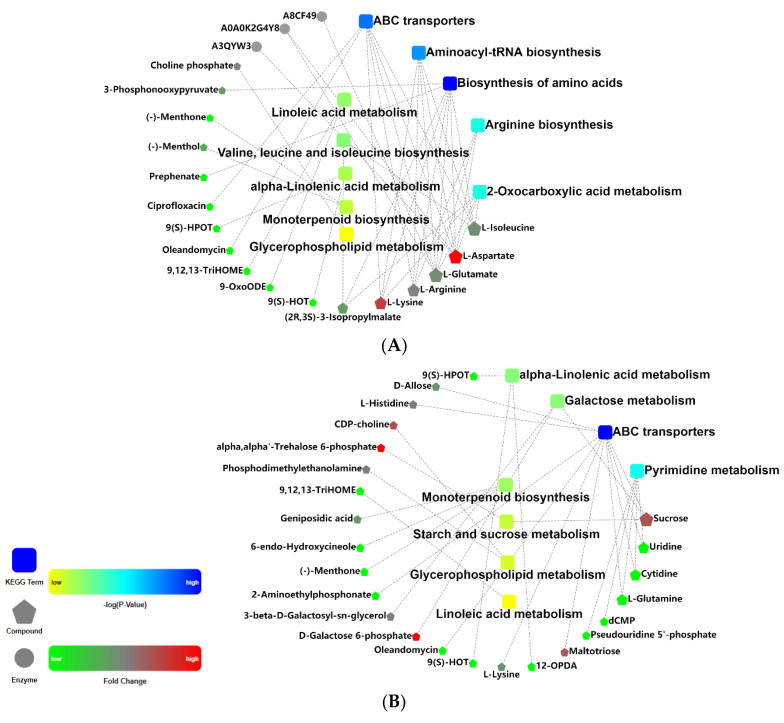
Metabolic network analyses of T2/T1 (**A**) and T3/T1 (**B**) samples using OmicsBean online software (http://www.omicsbean.cn, accessed on 12 June 2021). In yellow and blue area, rectangles represent -log (*p*-value) of KEGG pathways. In green and red area, pentagons represent values of fold change of differential metabolites. Round balls represent enzymes.

To further clarify the metabolic mechanism, the interrelation of the significantly enriched metabolic pathways, enzymes, and compounds were analyzed using OmicsBean online software (http://www.omicsbean.cn, accessed on 12 June 2021). As can be seen from the network analysis (Figure 6), the number of significantly different KEGG pathways, enzymes and metabolites, and levels of metabolites were significantly different in the two comparison groups. For the T2/T1 comparative group, except prephenate, the levels of most compounds (L-glutamate, L-lysine, L-aspartic acid, L-arginine, L-isoleucine, phosphohydroxypyruvic acid, 3-isopropylmalate and (S)-2-aceto-2-hydroxybutanoic acid) were increased in biosynthesis of amino acids. Generally speaking, biosynthesis of amino acids plays a vital role in response to heavy metal stress of plants through adjusting osmotic pressure, accumulating compatible osmotic agents, regulating intracellular pH, and detoxicating ROS [66]. It was shown that Cd stress resulted in remarkable variations on biosynthesis of amino acids, sugar, and organic acids in radish roots [67]. As depicted in Figure 5 and Figure 6, differential metabolites were significantly enriched in biosynthesis of amino acids in T2/T1samples (48 h Cd stress). Metabolomic analysis demonstrated that radish roots and *Elodea nuttallii* shoots exposed to Cd caused significant changes in biosynthesis of amino acids and aminoacyl-tRNA biosynthesis [36,67]. Quantitative proteomic analysis demonstrated that differentially expressed proteins of *Iris lactea* var. *chinensis* in response to Cd stress are mainly involved in biosynthesis of amino acids [68]. Campos et al. [66] revealed that *Pityrogramma calomelanos* responded to arsenic (As) stress by enhancing the biosynthesis of amino acids through metabonomic analysis. The differentially expressed genes were enriched in biosynthesis of amino acids in Indian mustard under As stress [69].

Alpha-linolenic acid metabolism also plays an important role in biotic and abiotic stress. A present study found that the expression of alpha linolenic acid metabolism related genes in soybean response to soybean cyst nematode changed significantly [70]. Zhang et al. [71] reported that proteins responded to salt stress were enriched in the alpha-linolenic acid metabolism in sesame. In our present study, alpha-linolenic acid metabolism was enriched whether under 48 h or 7 d of Cd stress.

ABC transporters are critical for plants under adverse circumstance stress [72]. Cd can form complexes with glutathione (GSH) or phytochelatins (PC), which are then transferred to vacuoles by ABC transporters [73]. Therefore, ABC transporters have detoxification effects on a variety of heavy metals [74]. As illustrated in Figure 5 and Figure 6, differential metabolites were significantly enriched in ABC transporters in T2/T1 and T3/T1 samples. Levels of some amino acids—including L-glutamate, L-lysine L-aspartic acid, L-arginine, and L-isoleucine—were upregulated, while oleandomycin and ciprofloxacin were downregulated in ABC transporters under 48 h Cd stress. However, some amino acids (L-lysine, L-glutamine, and L-histidine) and carbohydrates (sucrose, D-maltose, allose and maltotriose) were upregulated, but oleandomycin, ciliatine were downregulated under 7 d Cd stress.

It has been reported that ABC transporter gene *PtoABCG36* was induced by Cd stress, and participated in the resistance to Cd stress. *PtoABCG36* could promote Cd tolerance by extracting it from the plants [72]. In addition, many ATP-binding cassette (ABC) transporter genes—such as *AtABCG22*, *ABCG34*, and *AtABCG16*—were induced rapidly under adverse circumstance stress [75,76,77]. Cao et al. [78] reported that the transmembrane transport of cellular material in response to Cd stress was mainly achieved by ABC transport in *Saliz matsudana*. Transcriptomic analysis showed that the expression of ABC transporters was significantly upregulated under the stress of Cd and Zn in wheat roots [79].

Additionally, variations in other pathways could also be made clear by their physiological functionalities. Glycerophospholipid metabolism, linolenic acid metabolism, and monoterpenoid biosynthesis also play crucial roles in biotic and abiotic stress [80,81,82]. These metabolic pathways were disturbed under 48 h and 7 d Cd stress in our present study. Yadav et al. [80] suggested that under As, Cd, and Cr stresses, differentially expressed genes of chickpea were enriched in glycerophospholipid metabolism using microarray analysis. Metabolome analysis based on LC-MS found that the differential metabolites of *Ganoderma lucidum* in response to Cd stress were significantly enriched in glycerophospholipid metabolism [83]. In addition, glycerophospholipid plays a pivotal role in maintaining the integrity of biomembrane. Mutation of some important genes (*CgHOG1* and *CgRDS2*) in glycerophospholipid metabolism could significantly decrease the cell growth and survival rate of *Candida glabrata* under salt stress [84]. Multiple-omics (transcriptomics, proteomics, and metabolomics) methods revealed that glycerophospholipid metabolism in *Saccharomyces cerevisiae* was significantly upregulated from the mRNA, protein, and metabolites levels to response hypoxic stress. Moreover, some metabolites (phosphatidylcholine, phosphatidylethanolamine, phosphatidic acids, and phosphoinositide) of glycerophospholipid metabolism played crucial roles in maintaining the stability of the cell membrane to reduce cell damage [85]. Chromium (Cr) stress upregulated genes involved in linolenic acid and arginine metabolism in *Brassica napus* by using transcriptome analysis [81]. Proteomic analysis showed that some differentially expressed proteins in soybean were enriched in linolenic acid metabolism to resist *Lamprosema indicate*, they played a defensive role under insect stress [86]. Some monoterpenes directly or indirectly protect against herbivores and pathogens in plants [87]. It was recently reported that monoterpenoid biosynthesis was remarkably enriched in rhizosphere microbiomes of spinach (*Spinacia oleracea* L., cv. Racoon) under salinity and drought [82]. Recently, Weinblum et al. [88] observed that the differentially expressed genes were enriched in monoterpenoid biosynthesis in response to the two-spotted spider mite (TSSM) infestation in tomato plants.

Thus, it was inferred that Cd tolerance of *B. juncea* roots under different time stress was regulated primarily by these metabolic pathways. Moreover, there were great differences in the enriched metabolic pathways under 48 h and 7 d Cd stress in *B. juncea* roots. Taken together, the interaction of metabolites, KEGG metabolic pathways, and enzymes coordinately regulate the response mechanism of *B. juncea* roots under Cd stress at different times.

## 3. Materials and Methods

### 3.1. Plant Material and Growth Conditions

Seeds of a *B. juncea* inbred line were surface sterilized, germinated in Petri dishes containing deionized water, and incubated in the dark at 25 °C for two days. Following germination, seedlings were transferred to plastic case filled with 500 mL of 1/2 Hoagland nutrient solution. The experiment was carried out in a greenhouse, where the day/night temperature and humidity regime of 25/20 °C and 55/75% relative humidity (RH), respectively. The daily light cycle was 16 h. The nutrient solution was continuously oxygenated and replaced every three days. After 40 days of Cd-free growth, the seedlings were randomly assigned to 80 µM CdCl_2_ with 1/2 Hoagland nutrient solution for 0 h (T1, control, CK), 48 h (T2), and 7 d (T3), respectively. At harvest, young roots (0–7 cm) for metabolomic analyses were immediately frozen in liquid nitrogen.

### 3.2. Chemicals

All chemicals and solvents are analytical or chromatographic grade in LC-MS analysis. Methanol, formic acid, water and acetonitrile were purchased from CNW Technologies GmbH (Düsseldorf, Germany). Internal standards for LC-MS, 2-chloro-l-phenylalanine and Lyso PC (17:0) were purchased from Shanghai Hengchuang Biological Technology Co., Ltd. (Shanghai, China) and Avanti Company (Alabaster, Alabama, USA), respectively. The Cd standard solution (China national standard sample no. GSB04-1721-2004) used in atomic absorption spectrometry assay was obtained from Guobiao (Beijing) Testing & Certification Co., Ltd. (Beijing, China). Hoagland nutrient solution was purchased from Qingdao Hope Bio-Tcehnology Co., Ltd. (Qindao, China). Evans blue was purchased from Sinopharm Chemical Reagent Co., Ltd. (Shanghai, China).

### 3.3. Measurements of Cd Content and Growth Parameters of Plant

The cadmium standard curve (Abs = 0.46900C + 0.019858, R^2^ = 0.997) was plotted using the Cd standard solution. *B. juncea* roots and leaves were prepared and digested according to the approach of Jiang et al. [40] and the Cd content was determined by Thermo Scientific iCE 3000 Series AA Spectrometers with three biological replicates. Thirty plants were randomly selected from each treatment group to measure the shoot height and root length with a ruler according to Ling et al. [89]. The dry weights of aboveground and underground parts were measured respectively according to Matse et al. [90]. Cut off the plant at the base of the stem with a blade and put it into an oven to dry to constant weight. Use an analytical balance to weigh the dry weight of the aboveground part and the underground part, respectively. The cell activity of root tip was determined by Evans blue staining according to Liu and Lin [91]. The root tip material was stained with 0.25% (*w*/*v*) Evans blue for 24 h, and photo-graphed under stereometric microscope. The data were analyzed by ANOVA using origin 9.1 software. The differences at *p* < 0.05 were shown to be significant by the LSD test.

### 3.4. Metabolites Extraction

Eighty mg *B. juncea* samples were accurately weighed and transferred to a 1.5 mL Eppendorf tube with two small steel balls. 2-chloro-l-phenylalanine (0.3 mg/mL, 20 μL) and Lyso PC (17:0) (0.1 mg/mL, 20 μL) dissolved in methanol (as internal standard), and 1 mL methanol-water solution (7/3, *v*/*v*) were added to each *B. juncea* sample. The samples were precooled at −20 °C for 2 min and then ground at the frequency of 60 Hz for 2 min in a TissueLyser. The mixtures were extracted for 30 min in an ultrasonic device at ambient temperature, and placed at −20 °C for 20 min. The extracts were centrifuged at 13,000 rpm for 10 min at 4 °C, and 300 μL of supernatant were collected and dried. 400 μL methanol-water solution (1/4, *v*/*v*) were added to each sample, and samples were extracted by ultrasonication for 2 min after vortexing, then centrifuged at 13,000 rpm for 10 min at 4 °C. 150 μL of supernatant was collected, followed by filtered using microfilters (0.22 μm) into LC vials. The filtrate was kept at −80 °C for further LC-MS analysis. Quality control (QC) samples were prepared by mixing aliquots of extracts from each sample to form a pooled sample, and the volume of each QC is the same as the sample.

### 3.5. LC-MS Analysis

The ACQUITY UHPLC system (Waters Corporation, Milford, MA, USA) coupled with an AB SCIEX Triple TOF 5600 system (AB SCIEX, Framingham, MA, USA), was applied to analyze the metabolic profiling in both positive and negative ionization modes as described by Xiong et al. [92]. In both positive and negative modes, the metabolite separation was conducted on an ACQUITY UPLC BEH C18 column (1.7 μm, 2.1 × 100 mm) at 40 °C (column temperature) with a flow rate (350 μL/min). The injection volume was 5 μL. The mobile phase consisted of (A) water (containing 0.1% formic acid, *v*/*v*) and (B) acetonitrile (containing 0.1% formic acid, *v*/*v*). The linear gradient elution program was shown in Table 1.

The positive and negative modes parameters of mass spectrometry were as follows: ion spray voltage 3500 V, capillary temperature 320 °C, heater temperature 350 °C, sheath gas flow rate 40 arb, aux gas flow rate10 arb, and s-lens RF level 50%. The range of reference masses (*m*/*z*) was set as 100–1000. In our experiments, six biological replicates were set up per *B. juncea* sample to LC-MS analysis. To appreciate the stability of the LC-MS system and reliably correct the repeatability of the data throughout the analysis, the QCs were injected at regular intervals (every six samples).

### 3.6. LC-MS Data Analyses

The obtained LC-MS raw data were analyzed using the progqenesis QI v2.3 software (Nonlinear Dynamics, Newcastle, UK) to make meaningful data mining, performing peak alignment, picking, normalization, and retention time correction. The parameters were set as follows: precursor tolerance 5 ppm, product tolerance 10 ppm, and production threshold 5%. The resulting matrix of features included information on mass-to-charge ratio (*m*/*z*), retention time (RT), and peak intensities. Metabolites were identified by progenesis QI Data Processing Software based on precise mass number, secondary fragments and isotope distribution, and qualitative by mapping on public databases such as HMDB (http://www.hmdb.ca/, accessed on 12 June 2021), Metlin (http://metlin.scripps.edu/, accessed on 12 June 2021), Lipidmaps (http://www.lipidmaps.org/, accessed on 12 June 2021), and self-built databases [93]. The data in both positive and negative modes were merged to form a combined data set, which was imported into R software package according to the method of Luo et al. [94]. Principle component analysis (PCA) and (orthogonal) partial least-squares-discriminant analysis (O) PLS-DA were performed to distinguish differences of metabolic profiles among experimental groups. Generally, these parameters, such as R^2^X, R^2^Y, and Q^2^, are used to assess the quality and reliability of established models. The model is considered as excellent fitness and predictive capability when three parameters (R^2^X, R^2^Y, and Q^2^) close to 1.0 [95]. Differential metabolites between groups were selected using multi-dimensional couple with single-dimensional analysis. In OPLS-DA analysis, variable important in projection (VIP) was used to discover differential metabolites with biological significance [96]. Furthermore, the significance of differential metabolites was further verified by the Student’s *t*-test. *B. juncea* metabolites with VIP > 1.0 and *p* < 0.05 were selected as differential metabolites [17].

### 3.7. Metabolic Pathways Enrichment Analysis

To reveal the mechanism of metabolic pathway variation in different samples, the differential metabolites were carried out metabolic pathway enrichment analysis based on the KEGG database (http://www.kegg.jp/kegg/pathway.html, accessed on 12 June 2021) [97]. Their KEGG ID and pathway were found, then the number of metabolites enriched in corresponding pathway was calculated. The pathway with *p*-value ≤ 0.05 was selected as enriched pathway, its calculation formula is

$$P=1-\sum_{i=0}^{m-1}\frac{\left(\begin{array}{c} M\\ i \end{array}\right)\left(\begin{array}{c} N-M\\ n-i \end{array}\right)}{\left(\begin{array}{c} N\\ n \end{array}\right)}$$

N: The total number of metabolites, n: The number of differential metabolites, M: the number of metabolites annotated as a specific pathway, m: the number of differential metabolites annotated as a specific pathway.

## 4. Conclusions

This paper investigated the physiological effects and the metabolic profiling of *B. juncea* exposed to Cd at different times using LC/MS-based metabolomics. Most of Cd absorbed by *B. juncea* was accumulated in the roots, and the damage of cells in root tips became more serious with longer Cd-stressed time. Compared with the control, 183 and 219 significantly differential metabolites were identified in *B. juncea* roots under 48 h and 7 d Cd stress, respectively. The metabolic profiling suggested these metabolites (mainly amino acids, organic acids, carbohydrates, lipids flavonoids, alkaloids, indoles, etc.) were caused great variations when exposed to Cd at different times in *B. juncea* roots. KEGG pathway analysis indicated that Cd stress resulted in significant changes (*p* < 0.01) involved in 8 (48 h) and 4 (7 d) metabolic pathways, respectively. These enriched metabolic pathways and significantly differential metabolites could be the main point of adjustment for elevating the enrichment or tolerance of *B. juncea* to Cd. These findings will not only help to know its mechanism of Cd resistance, but also further improve the remediation efficiency of Cd-polluted environment.

## Figures and Tables

**Table 1:** Elution gradient.

Time (min)	Mobile Phase Composition	
	A (%)	B (%)
0	95	5
1.5	95	5
3	70	30
7	40	60
9	10	90
11	0	100
13	0	100
13.2	95	5
16	95	5

## Data Availability

The data presented in this study are available on request from the corresponding author.

## Supplementary Materials

The following are available online at https://www.mdpi.com/article/10.3390/metabo11060383/s1, Figure S1: Base peak chromatogram of the T1 (A), T2 (B), and T3 (C) samples in positive ESI mode, Figure S2: Base peak chromatogram of the T1 (A), T2 (B), and T3 (C) samples in negative ESI mode, Figure S3: PCA score scatter plots of LC−MS data from six biological replicates of each *Brassica juncea* sample, Figure S4: Multivariate statistical score scatter plots and permutation test between T2 and T1, Figure S5: Multivariate statistical score scatter plots and permutation test between T3 and T1, Table S1: The differential metabolites between T2 and T1, Table S2: The differential metabolites between T3 and T1.
