# Supplementary material for: Metabolic Profiles of Brassica juncea Roots in Response to Cadmium Stress
Source: Metabolites. 2021 Jun 13;11(6):383. doi: 10.3390/metabo11060383 (PMC8232002; doi:10.3390/metabo11060383)
Supplement: Supplementary file 1 [file metabolites-11-00383-s001.zip › Fig.S1-S5.pdf]

(A)

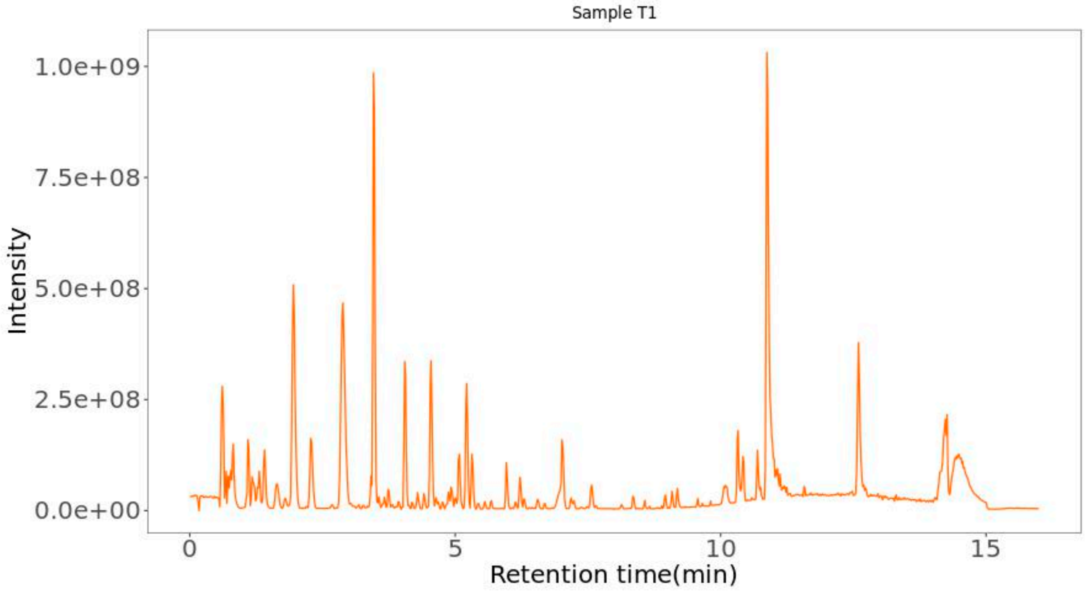

Sample T1
1.0e+09
7.5e+08
5.0e+08
2.5e+08
0.0e+00
Intensity
0
5
10
15
Retention time(min)

(B)

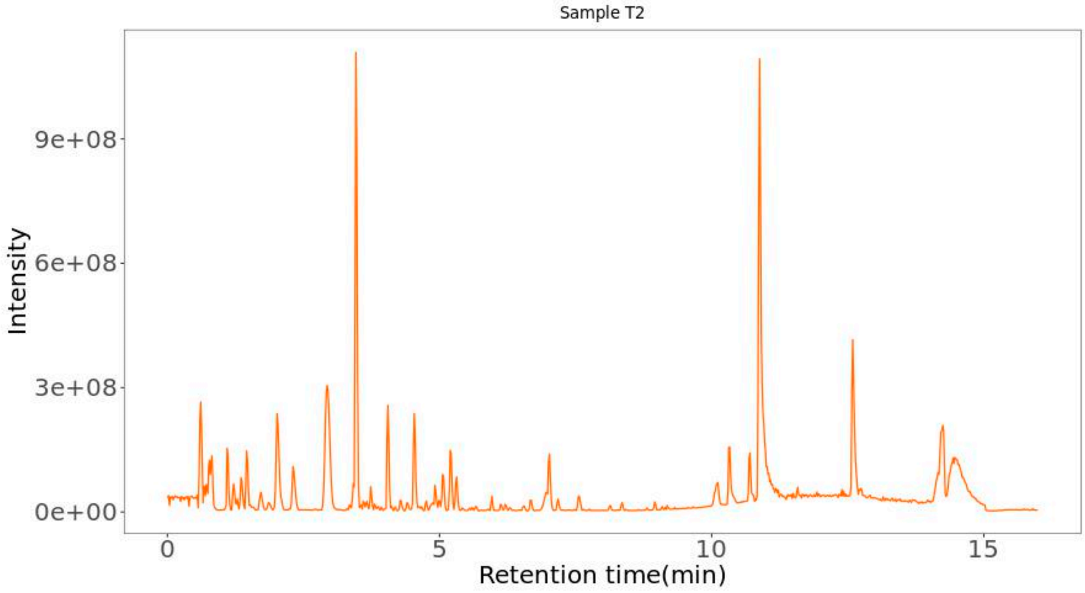

Sample T2
9e+08
6e+08
3e+08
0e+00
Intensity
0
5
10
15
Retention time(min)

(C)

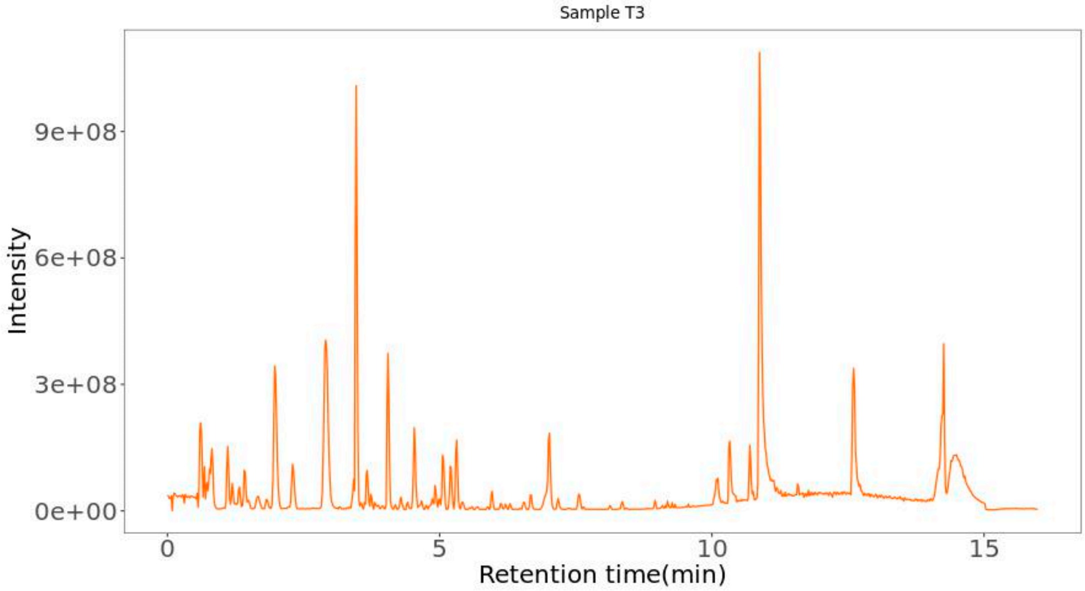

Fig. S1. Base peak chromatogram of the T1 (A), T2 (B), and T3 (C) samples in positive ESI mode.

(A)

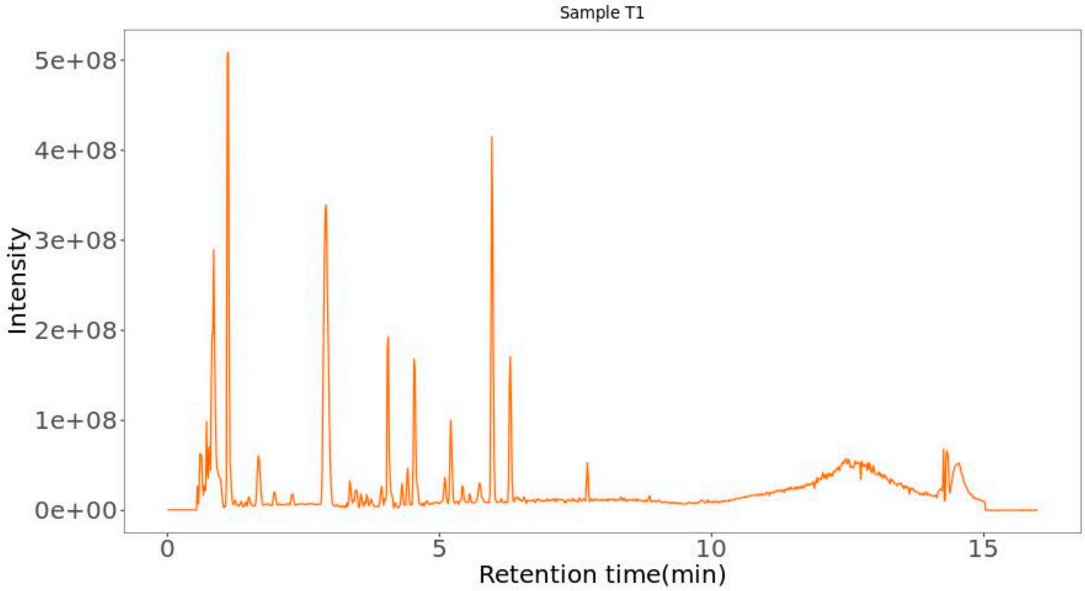

(B)

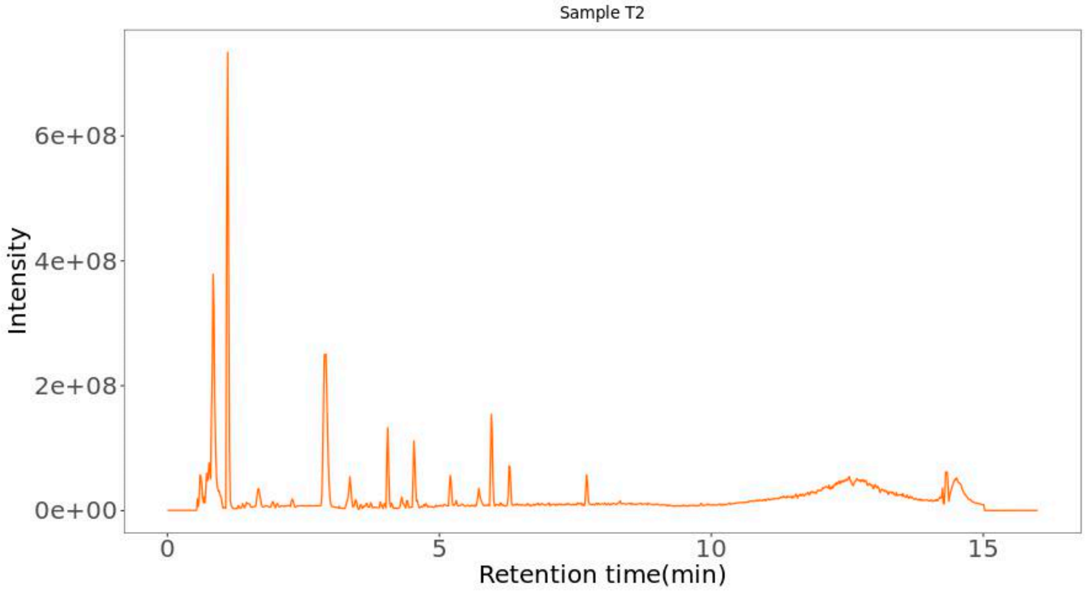

(C)

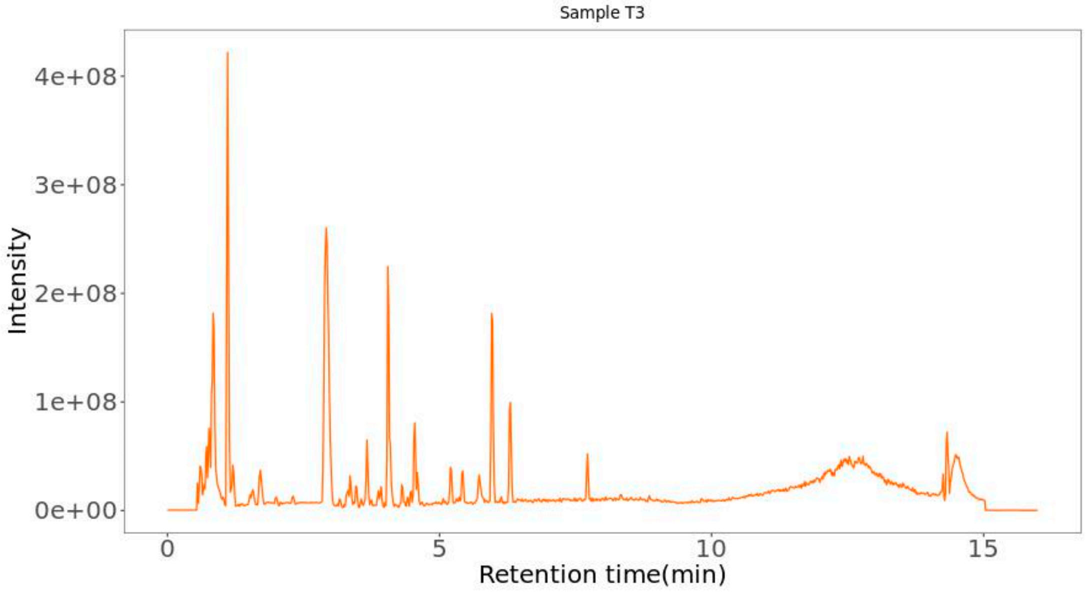

Fig. S2. Base peak chromatogram of the T1 (A), T2 (B), and T3 (C) samples in negative ESI mode.

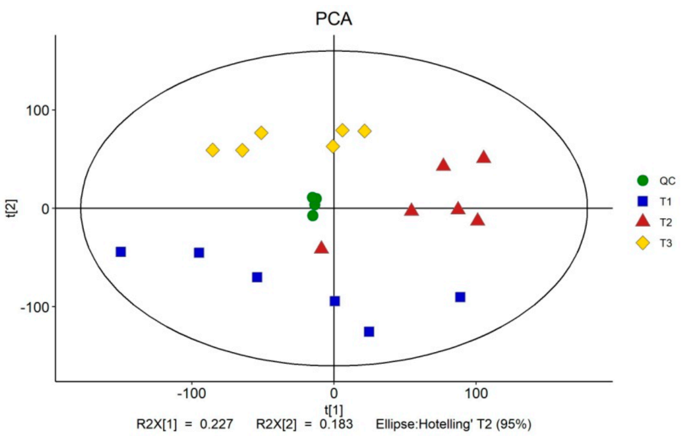

Fig. S3. PCA score scatter plots of LC−MS data from six biological replicates of each *Brassica juncea* sample.

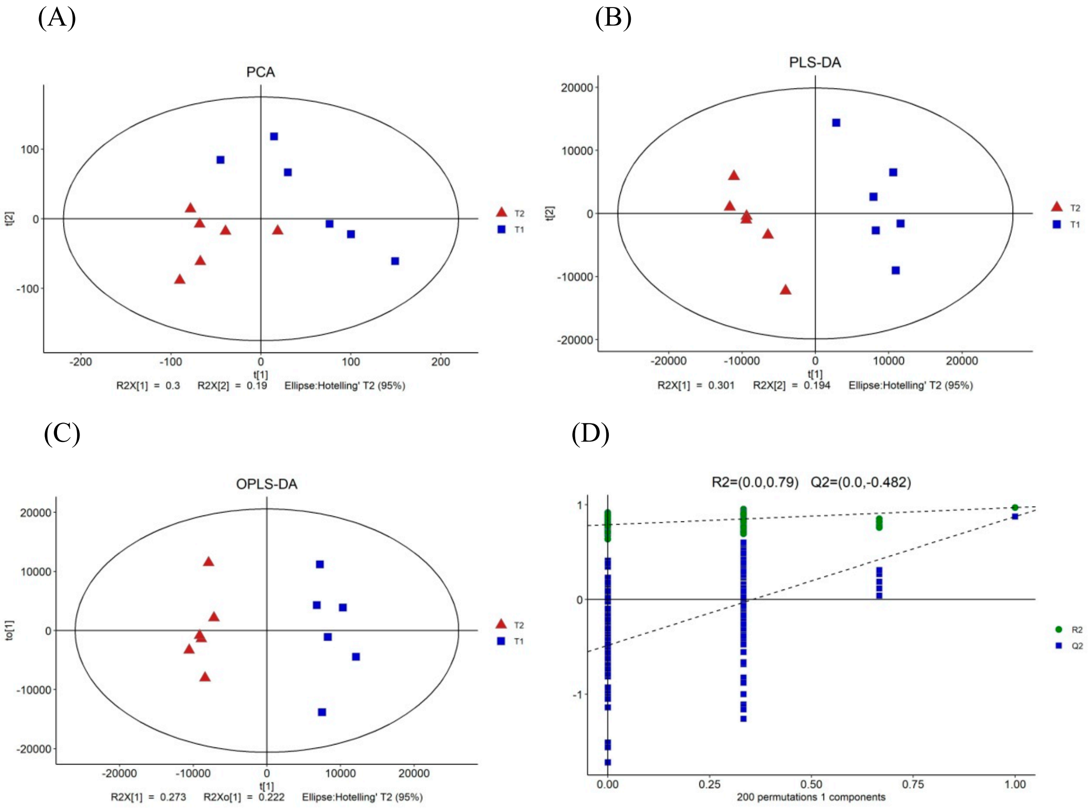

Fig. S4. Multivariate statistical score scatter plots and permutation test between T2 and T1. (A) PCA; (B) PLS-DA; (C) OPLS-DA; (D) permutation test of the OPLS-DA model.

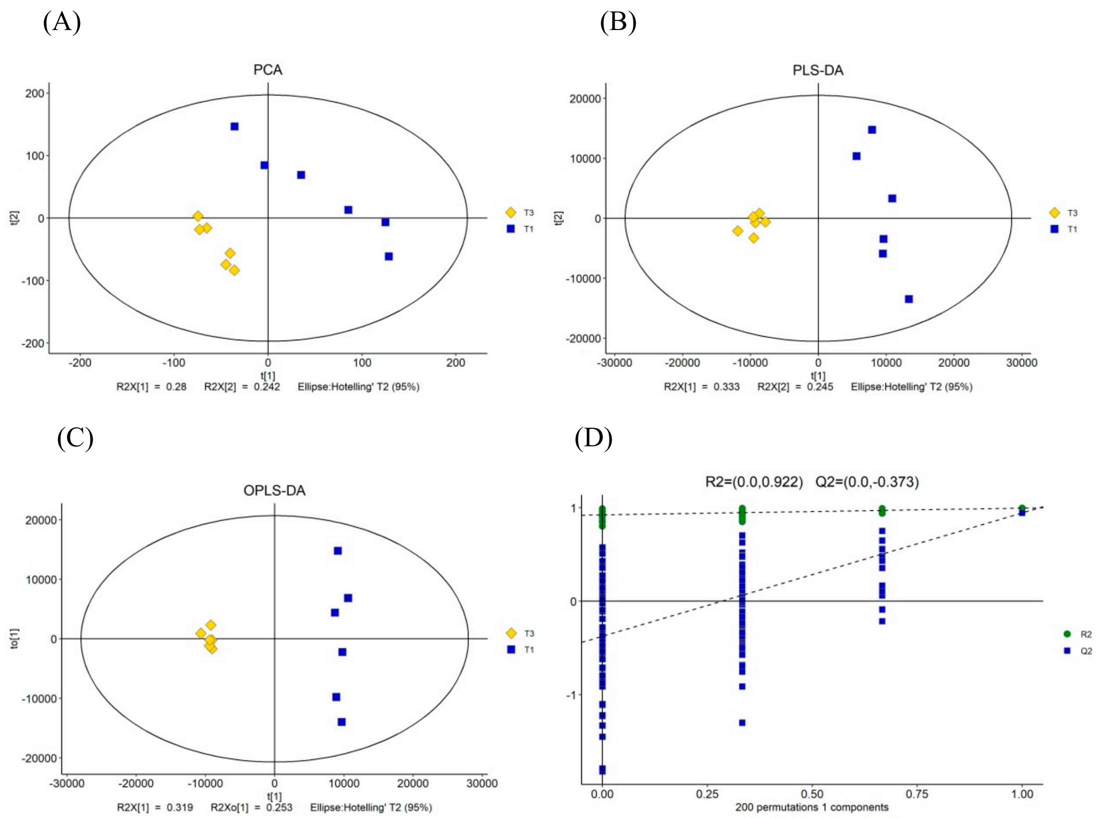

Fig. S5. Multivariate statistical score scatter plots and permutation test between T3 and T1. (A) PCA; (B) PLS-DA; (C) OPLS-DA; (D) permutation test of the OPLS-DA model.
